# Triphasic waves in electroencephalogram as a possible early marker of carcinomatous meningitis: a case report

**DOI:** 10.1097/MD.0000000000021735

**Published:** 2020-08-14

**Authors:** Chang Liu, Shihuan Cheng, Yue Ma, Caiyun Liu, Yudan Lv

**Affiliations:** a Department of Neurology and Neuroscience Center; b Department of Rehabilitation; c Department of Radiology, The First Hospital of Jilin University, Changchun, China.

**Keywords:** carcinomatous meningitis, electroencephalogram, triphasic waves

## Abstract

**Rationale:**

Carcinomatous meningitis is a rare neurological complication. This condition is difficult to diagnose, and misdiagnosis is common because the clinical manifestations are variable. Cerebrospinal fluid (CSF) cytology is the gold standard for diagnosis. Repeated lumbar puncture is required because of the low positive rate. Our case showed triphasic waves (TWs) in an electroencephalogram (EEG) before cancer cells were detected in cytology. We report this case to demonstrate that TWs in EEG may be a prognostic marker in patients with carcinomatous meningitis.

**Patient concerns:**

A 76-year-old Chinese male displayed incremental headache, nausea, emesis, and intermittent fever for 2 months. A routine scalp EEG showed mild slow background activity. The CSF analysis demonstrated a slight increase in protein, and the white blood cell count was in the normal range. Cytology did not show any atypical cells. Viral meningitis was considered for the first time.

**Diagnosis:**

After admission, a long-term EEG was performed because of his fever and mild abnormalities in the routine EEG. The second EEG showed asymmetric TWs in the frontal brain regions. Lung adenocarcinoma was found after systemic investigation. Finally, the patient was diagnosed with carcinomatous meningitis based on repeated CSF cytology.

**Interventions:**

The patient received systemic chemotherapy in the Department of Oncology.

**Outcomes:**

The patient was followed up monthly, and he was lost to follow-up in the sixth month after carcinomatous meningitis was diagnosed.

**Lessons:**

It is difficult to make a diagnosis in the early stage of carcinomatous meningitis because the clinical manifestations lack specificity. Repeated lumbar puncture is time consuming and is painful for the patients. In our case, TWs in EEG were detected before cancer cells were found in cytology. EEG should be performed when carcinomatous meningitis is under consideration.

## 1 Introduction

Carcinomatous meningitis is a neurological complication that occurs in cancer patients. It is difficult to make a diagnosis because the clinical manifestations are variable and include varying degrees of headache, cognitive symptoms, speech disorders, and convulsions.^[1]^ This condition may be misdiagnosed because some of the manifestations are atypical and lack specificity. A reliable method of diagnosis is cancer cells that are found in the cerebrospinal fluid (CSF).^[2]^ A single CSF examination of cytology has a low positive rate, so early diagnosis is difficult.

Triphasic waves (TWs) are form descriptions in electroencephalogram (EEG). Classical TWs show widespread synchronization and are composed of 3 parts: the first part is a small negative sharp wave, the second part is an obvious positive sharp wave, and the third part is a negative slow wave. These waves are found in metabolic encephalopathy, especially hepatic or renal diseases.^[3]^ The probable pathogenesis is the dysfunction of the oscillatory system between the cortex and thalamus. This phenomenon is also reported in other diseases, such as toxic encephalopathy, anoxic encephalopathy, Creutzfeldt-Jakob disease, and stroke.^[4]^ However, TWs have rarely been reported in carcinomatous meningitis.

We reported a case of TWs on EEG that was diagnosed as carcinomatous meningitis. Finally, we aimed to highlight TWs as a probable early diagnostic marker of carcinomatous meningitis.

## 2 Case report

**Figure 1:**
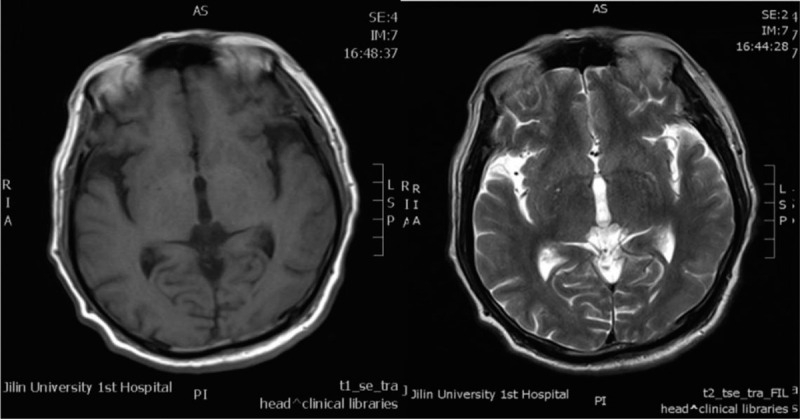
Patient's axial T1 and T2 weighted images showing lacunar infarction in the basal ganglia region.

**Figure 2:**
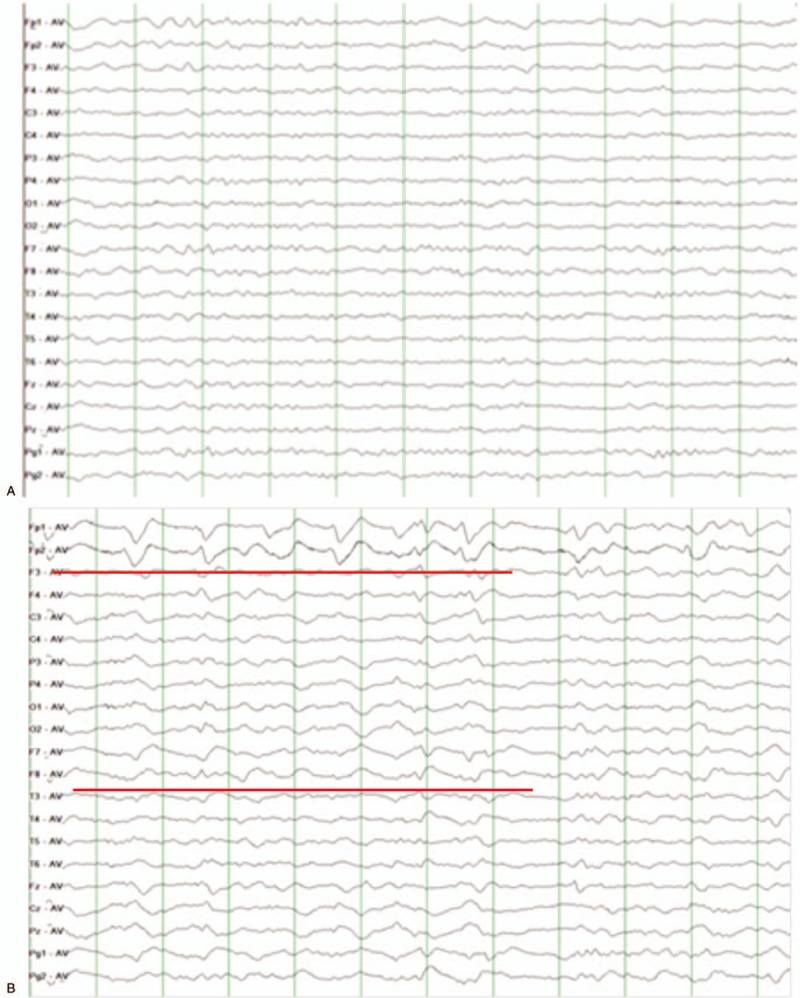
(A) The first EEG showing mild slow background activity without discharge and focal slow activity. (B) The second EEG showed a marked slow background, with 1 to 1.5 Hz periodic triphasic waves occurring in both the frontal and anterior temporal regions (HF 70 Hz, LF 0.5 Hz, sensitivity 10 μV/mm). EEG = electroencephalogram.

**Figure 3:**
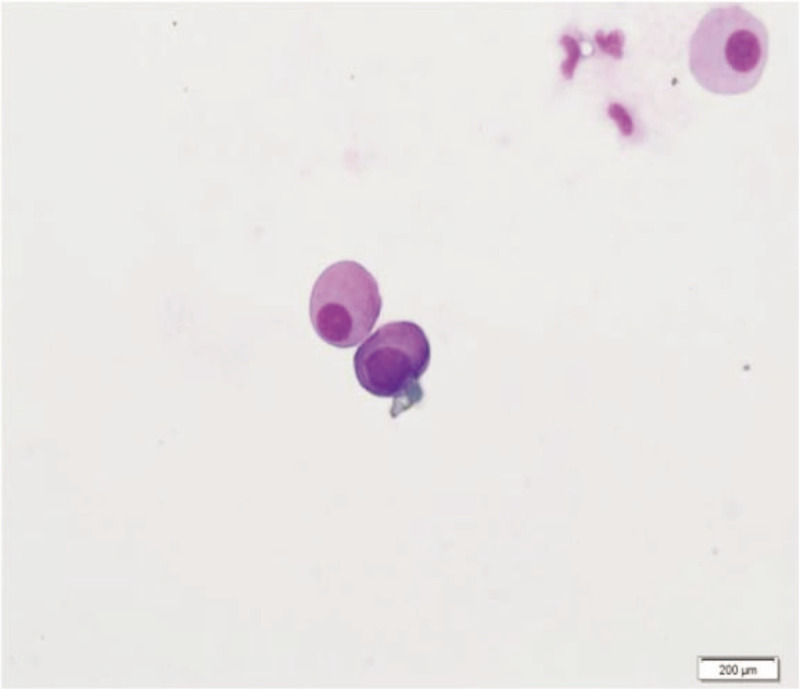
CSF cytology showed that abnormal cells had a larger cell area and nucleocytoplasmic ratio than monocytes and enlarged and hyperchromatic nuclei. Giemsa stain; ×200 magnification. CSF = cerebrospinal fluid.

A 76-year-old man reported incremental headache, nausea, emesis, and intermittent fever for 2 months. He had no previous organic brain diseases but had a long history of heavy smoking. Neurological examination showed negative findings but cervical rigidity. Magnetic resonance imaging (MRI) of the brain showed unremarkable abnormalities except for some small lacunar infarctions in the basal ganglia region (Fig. 1). Routine scalp EEG showed mild slow background activity (basic rhythm 7–8 Hz), but neither discharge nor focal slow activity nor underlying seizures were found (Fig. 2A). Blood tests showed a raised white blood cells (WBC) count of 18 × 10^12^ cells/L (reference range 4–10 × 10^12^ cells/L) and a mildly decreased hemoglobin level of 96 g/L (reference range 120–160 g/L); hepatic and renal function tests were all within the normal range. The CSF pressure was 220 mmH2O, the protein level was slightly increased at 0.76 g/L (reference range 0.15–0.45 g/L), the WBC count was 6 × 10^6^ cells/L (reference range 0–6 × 10^6^ cells/L, the proportion of mononuclear cells was 0.8, and that of multinuclear cells was 0.2), and glucose and chlorides were within the normal range. Cytology did not find any atypical cells. The patient was considered to have meningitis and admitted to the Department of Neurology. Five days after admission, a long-term EEG was performed because of his fever and mild abnormalities in the routine EEG. The second EEG showed a substantially slowed background activity without a normal rhythm, and asymmetric triphasic complex sharp waves were found in frontal brain regions (Fig. 2B), but no underlying seizure was detected. Subsequently, a systemic investigation was performed, and a lung computed tomography scan showed a nodule in the upper lobe of the right lung. Adenocarcinoma cells were found by exfoliative cytologic examination of sputum. Non-small cell lung cancer was diagnosed (stage IVB, T1bN2M1c). A second cytologic examination of CSF was performed, and abnormal cells were found. These cells had larger cell areas and nucleocytoplasmic ratios than monocytes and enlarged and hyperchromatic nuclei (Fig. 3). Therefore, the patient was diagnosed with carcinomatous meningitis. The patient received systemic therapy in the Department of Oncology. He received whole brain radiotherapy and intrathecal injection of methotrexate once a week in the first month then intrathecal injection monthly thereafter. The clinical symptom (headache and vomiting) relieved after the third month. The patient was lost to follow-up in the sixth month.

## 3 Discussion

Carcinomatous meningitis is a neurological complication that occurs in cancer patients. This condition is difficult to diagnose because the clinical manifestations are variable and atypical in some cases. They may be mistaken for meningitis because of the symptoms of headache, fever, vomiting, and stiff neck. The diagnostic management of carcinomatous meningitis is based on cytology and other investigations, such as MRI of the primary tumor or positron emission tomography-computed tomography of the whole body. A reliable method of diagnosis is cancer cells found in the CSF.^[5]^

CSF examination includes routine tests, biochemistry and cytology. Routine tests are always nonspecific. In carcinomatous meningitis patients, the incidence of increased CSF pressure is 50% to 70%, which is always increased slightly or substantially. Increased protein is found in 70% to 80% of cases and decreased glucose in 35% to 50% of cases.^[6]^ Cancer cells found in the CSF are the most valuable evidence for the diagnosis of carcinomatous meningitis. The probability of finding cancer cells is approximately 50% by a single cytology analysis and up to 90% with 3 analyses.^[7]^ MRI scan manifestations include diffuse pia mater enhancement, obstructive hydrocephalus, or no abnormalities. This condition can be confused with bacterial or fungal meningitis.^[8]^

Our patient's initial presentations were headache, vomiting, and fever. He had a slightly increased CSF pressure, increased protein, and normal glucose. The WBC count of the CSF was within the normal range, but inflammatory cells were found. We did not find any abnormal cells the first time. Routine EEG showed a slightly slow background without epileptic discharge and seizure. Therefore, the patient was first considered to have meningitis. However, the previous diagnosis should be questioned because of a second EEG manifestation that showed dominant TWs in the frontal brain regions. Based on experience, TWs are most commonly observed in patients with metabolic or toxic encephalopathy. Therefore, we performed a further investigation. Cancer cells were found in the CSF in the second lumbar puncture. In our case, TWs in EEG were detected earlier than positive results in cytology. There are few reports of the characteristic EEG pattern, and the relationship between the form of TWs in EEG and carcinomatous meningitis should be investigated.

The classical TW is 1.5 to 2.5 Hz, which can be observed most conspicuously on bipolar electrode lead. A majority of TWs show widespread synchronization. General TWs are considered to be linked with metabolic and toxic encephalopathy, such as hepatic or renal diseases, hyponatremia, hypercalcemia, hyperthyroidism, hyperglycemia, and hypoxic-ischemic encephalopathy. Focal TWs are present mainly in the frontal region and the occiput.^[9]^ Typical and characteristic EEGs in Creutzfeldt–Jakob disease are bisynchronous periodic waves. Focal or unilateral TWs were found in stroke, viral encephalitis, and dementia.^[10]^ We reviewed a case report featuring rapidly progressive dementia. The EEG showed periodic biphasic and triphasic complex sharp waves at 0.5 to 1.5 Hz in both frontotemporal leads. CSF cytology showed eccentric nuclei and deeply basophilic cytoplasm.^[11]^ In our case, the TWs were asymmetric in the frontal region. Dementia plays a role in the formation of periodic and symmetric TWs in the previous case. The mechanisms of the formation of TWs are not completely known. The probable pathogenesis is dysfunction of the oscillatory system between the cortex and thalamus for metabolic encephalopathy. Extensive structural or metabolic injuries of the brain stem, diencephalon, and subcortex may be caused by nonmetabolic factors. Some studies have shown that TWs may be correlated with intracranial hypertension.^[12]^ Changes in cerebral brain flow may influence the frequency of EEG. Lower cerebral brain fluid decreases fast waves and increases slow waves.^[13]^ For carcinomatous meningitis, cancer cells disseminate diffusely on the leptomeninges or cerebral cortex. Synaptic connections between neurons of the cortex are destroyed by cancer cells, and cortex activities are inhibited. CSF dynamic changes are due to cancer cells. These factors probably lead to a triphasic EEG pattern.

EEG is a noninvasive tool for detecting early-stage disease due to its high sensitivity. TWs can be found in patients with carcinomatous meningitis, although EEG is not a routine examination for these patients. This case suggests that TWs can be found in the early stage of carcinomatous meningitis, and carcinomatous meningitis should be considered when the TW pattern is shown in EEG.

## Author contributions

**Conceptualization:** Chang Liu, Yudan Lv.

**Data curation:** Yue Ma.

**Software:** Caiyun Liu.

**Supervision:** Yudan Lv.

**Writing – original draft:** Chang Liu.

**Writing – review & editing:** Shihuan Cheng.
